# The growth of non-solid neoplastic lung nodules is associated with low PD L1 expression, irrespective of sampling technique

**DOI:** 10.1186/s12967-020-02241-y

**Published:** 2020-02-03

**Authors:** Chandra Bortolotto, Claudio Maglia, Antonio Ciuffreda, Manuela Coretti, Roberta Catania, Filippo Antonacci, Sergio Carnevale, Ivana Sarotto, Roberto Dore, Andrea Riccardo Filippi, Gabriele Chiara, Daniele Regge, Lorenzo Preda, Patrizia Morbini, Giulia Maria Stella

**Affiliations:** 1 Department of Intensive Medicine, Unit of Radiology, IRCCS Policlinico San Matteo Foundation and University of Pavia Medical School, Pavia, Italy; 2 Radiology Unit, IRCCS Candiolo Cancer Institute and University of Turin Medical School, Candiolo, TO Italy; 3 Department of Medical Sciences and Infective Diseases, Unit of Respiratory Diseases, IRCCS Policlinico San Matteo Foundation and University of Pavia Medical School, 27100 Pavia, Italy; 4 Department of Intensive Medicine, Unit of Cardiothoracic Surgery, IRCCS Policlinico San Matteo Foundation and University of Pavia Medical School, Pavia, Italy; 5 Department of Molecular Medicine, Unit of Pathology, IRCCS Policlinico San Matteo Foundation and University of Pavia Medical School, Pavia, Italy; 6 Unit of Pathology, IRCCS Candiolo Cancer Institute, Candiolo, TO Italy; 7 Department of Medical Sciences and Infective Diseases, Unit of Radiation Therapy, IRCCS Policlinico San Matteo Foundation and University of Pavia Medical School, Pavia, Italy; 8 Department of Clinical-Surgical, Diagnostic and Pediatric Sciences, University of Pavia, Pavia, Italy

**Keywords:** Imaging, Biopsy, Nodule, Lung cancer, PD-L1, Molecular profiling

## Abstract

**Background:**

Few data are known regarding the molecular features and patterns of growth and presentation which characterize those lung neoplastic lesions presenting as non-solid nodules (NSN).

**Methods:**

We retrospectively reviewed two different cohorts of NSNs detected by CT scan which, after transthoracic fine-needle aspiration (FNA) and core needle biopsy (CNB) received a final diagnosis of malignancy. All the enrolled patients were then addressed to surgical removal of lung cancer nodules or to exclusive radiotherapy. Exhaustive clinical and radiological features were available for each case.

**Results:**

In all 62 analysed cases the diagnosis of adenocarcinoma (ADC) was reached. In cytologic samples, *EGFR* activating mutations were identified in 2 of the 28 cases (7%); no case showed *ALK/EML4* or *ROS1* translocations. In the histologic samples *EGFR* activating mutation were found in 4 out of 25 cases (16%). PD-L1 immunostains could be evaluated in 30 cytologic samples, while the remaining 7 did not reach the cellularity threshold for evaluation. TPS was < 1% in 26 cases,  > 1% < 50% in 3, and > 50% in 1. All surgical samples showed TPS < 1%. Of the 17 cases that could be evaluated on both samples, 15 were concordantly TPS 0, and 2 showed TPS > 1% < 50 on the biopsy samples. TPS was < 1% in 14 cases, > 1%/< 5% in 4 cases, > 5%/< 50% in 2 cases, > 50% in 1 case.

**Conclusions:**

Overall PD-L1 immunostaining documented the predominance of low/negative TPS, with high concordance in FNA and corresponding surgical samples. It can be hypothesized that lung ADC with NSN pattern and predominant in situ (i.e. lepidic) components represent the first steps in tumor progression, which have not yet triggered immune response, and/or have not accumulated a significant rate of mutations and neoantigen production, or that they belong to the infiltrated-excluded category of tumors. The negative prediction of response to immunomodulating therapy underlines the importance of rapid surgical treatment of these lesions. Notably, cell block cytology seems to fail in detecting *EGFR* mutations, thus suggesting that this kind of sampling technique should be not adequate in case of DNA direct sequencing.

## Background

Non-solid nodules (NSNs) are commonly detected at thoracic CT scan. They are identified as circumscribed areas of increased lung attenuation with preservation of the bronchial and vascular margins and are also referred to as a ground glass opacity [1]. A defined NSN can be either partly solid (part of the nodule completely obscures the underlying lung parenchyma) or pure non-solid [2]. They can be a manifestation of inflammation, infection, or fibrosis, but they can also be precursors of lung cancer, mainly adenocarcinoma (atypical adenomatous hyperplasia). Notably, persistent lesions have a high likelihood of malignancy. The ELCAP study reported a malignancy rate of 34% for all NSNs, 18% for pure ground glass lesions and 63% for partly solid lesions. Overall the latter featured even higher malignancy rates reaching up to 75% in other reports [3]. CT-guided needle biopsy of ground glass lesions is a useful diagnostic approach with acceptable complication rates approaching those of solid lesions [4–7]. It has been reported that in mixed nodules the diagnostic accuracy is influenced by the ground glass component, being lower in pure non-solid opacities [5].

**Fig. 1:**
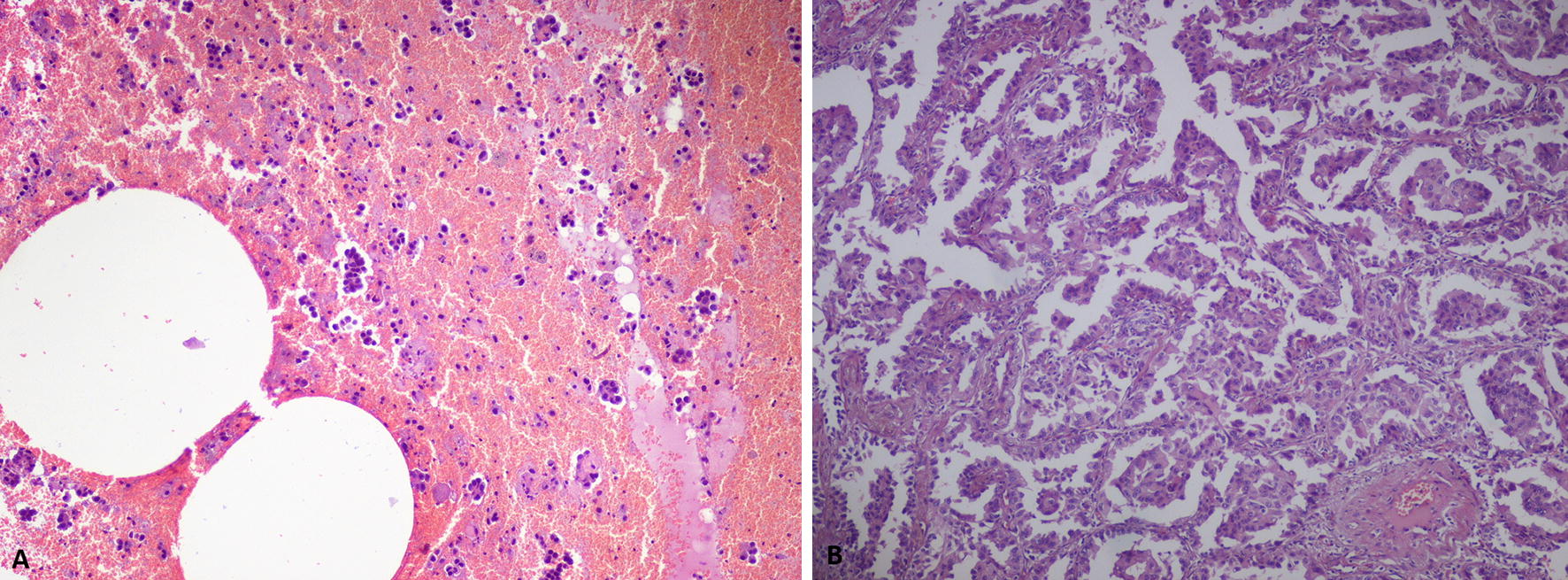
Morphology of non-solid nodules samples obtained through CT-guided biopsy. **a** Cell-block of CT-guided biopsy with neoplastic epithelial cell with secretive features, arranged in strains and micro-papillae (H&E, 100×). **b** Adenocarcinoma of the lung with prevalent lepidic growth. Invasive features are focally present in the neoplasia (H&E, 100x)

Personalized approach to lung cancer generally requires tissue samples in order to perform exhaustive screening of molecular defects in actionable target genes. We and others showed the feasibility of tumor mutational screening in aspirated lung cancer cells (fine needle aspiration, FNA) obtained through imaging guidance [8–10]. At the same time, many other data underlined reliability of core needle biopsy (CNB) for lung cancer molecular profiling, even if some reports documented a slightly higher rate of complications [11–13]. More recently, both FNA cytology and CNB have been reported to be accurate, feasible and safe procedures for PD-L1 testing both retrospectively and in prospective studies [14–16]. It should be underlined that few data are known on the molecular features which characterize neoplastic lesions presenting as NSN, and poor information are available regarding the specific pattern of tumour presentation and growth as well as the diagnostic accuracy of different bioptic approaches in these settings. To explore these issues, the present study is focused on the analysis of the radiological and molecular aspects of two series of non-solid neoplastic nodules obtained through two different transthoracic approaches and aimed at generating their matched radio-pathologic profiles. Moreover, we could compare the results obtained on cytological specimens with the subsequent corresponding resected surgical samples to validate the bioptic preliminary data (Fig. 1).

## Patients and methods

### Patients identification and selection

We retrospectively reviewed the CT scans of patients who were followed at IRCCS San Matteo Hospital Foundation and underwent transthoracic CT-guided fine needle aspiration of suspected pulmonary lesions from October 2016 to December 2017 to select the cases showing imaging features consistent with non-solid and mixed lung nodules. We identified 40 cases of which 37 subsequently received the diagnosis of malignancy, whereas for the remaining three cases the diagnosis was coherent with epithelioid aggregates (1 case), granulomas (1 case), sarcoidosis (1 case). Based on early disease stage, all the enrolled patients were then addressed to surgical removal of lung cancer nodules and for 20 out of the 37 analysed cases, the post-operative histology was available for analysis. To further validate this work, we also retrospectively reviewed the CT scans of patients followed at IRCCS Candiolo Cancer Institute, who underwent transthoracic CT-guided core needle biopsy of suspected pulmonary lesions from March 2016 to June 2018. During this temporal range, we identified 25 cases of NSNs that subsequently received the diagnosis of lung cancer. As above indicated, based on the different stages of presentation, the enrolled patients were then addressed to surgical removal of lung cancer nodules or to exclusive radiotherapy.

For each case exhaustive clinical, histological, molecular and procedural data were collected and available in Tables 1 and 2.

**Table 1:** Exhaustive radio-morphologic and molecular data on the evaluated NS nodules approached through CNB bioptic procedures

ID#	Gender	Age at diagnosis (yrs)	Lobe	Segment	Site	Diameter (mm)	Density M/NS	Histology	Architectural pattern	EGFR	ROS1	EML4/ALK	PDL-1 (%)
1	F	78	IRL	Posterior	P	61	M	Atypia/ADC	Atipic hyperplasia/ADC	wt	wt	wt	5
2	F	53	ILL	Lateral	P	26	NS	ADC	In situ, not mucinous	p.L861Q	wt	wt	3
3	F	72	SLL	Apico-posterior	P	33	M	ADC	Mucinous	wt	wt	wt	< 1
4	M	79	SRL	Posterior	P	30	M	ADC	In situ	wt	wt	wt	< 1
5	F	62	SRL	Apical	P	26	M	ADC	Papillary (10%) lepidic (20%) acinar (70%)	p. L858R	wt	wt	< 1
6	F	75	SLL	Apico-posterior	M	36	M	ADC	Lepidic/60%)	wt	wt	wt	< 1
7	F	80	SRL	Posterior	P	28	M	ADC	Acinar (60%) micropapillary (20%) solid (10%) lepidic (10%)	wt	wt	wt	< 1
8	F	46	SRL	Apical	M	15	NS	ADC	Lepidic (60%) acinar (40%)	wt	wt	wt	< 1
9	F	59	SRL	Apical	P	16	NS	ADC	Papillary (70%) Solid (30%)	p.G719A	wt	wt	5
10	M	69	SRL	Apical	P	30	NS	ADC	Well differentiated ADC	wt	wt	wt	20
11	F	69	SRL	Posterior	P	20	NS	ADC	Well differentiated ADC	wt	wt	wt	< 1
12	M	71	SRL	Apical	P	27	M	ADC	Partially differentiated ADC	wt	wt	wt	< 1
13	M	71	SRL	Anterior	M	25	M	ADC	Well differentiated ADC	wt	wt	wt	< 1
14	M	76	SLL	Apico-posterior	M	14	M	ADC	Acinar (90%) Lepidic (10%)	wt	wt	wt	< 1
15	M	58	IRL	Posterior	P	43	M	ADC	Acinar (60) Papillary (30%) Micropapillary (10%)	wt	wt	wt	< 1
16	F	81	SRL	Anterior	P	26	M	ADC	Well differentiated ADC	wt	wt	wt	52
17	M	71	SRL	Anterior	M	28	M	ADC	Well differentiated ADC	wt	wt	wt	< 1
18	M	77	SRL	Posterior	P	22	NS	ADC	Acinar (80%) Lepidic (20%)	wt	wt	wt	11
19	F	62	SRL	Posterior	M	16	NS	ADC	Partially differentiated ADC	wt	wt	wt	< 1
20	F	60	SLL	Superior lingular	M	30	NS	ADC	Lepidic (60%) acinar(40%)	wt	wt	wt	< 1
21	M	75	SRL	Posterior	P	35	NS	ADC	In situ, mucinous	wt	wt	wt	< 1
22	F	75	SRL	Apical	P	25	M	ADC	Partially differentiated ADC, anthracosis	wt	wt	wt	n.a
23	M	64	SRL	Apical	P	32	M	ADC	Well differentiated ADC	wt	wt	wt	3
24	F	53	SLL	Apico-posterior	M	24	NS	ADC	Well differentiated ADC	del ex 19	wt	wt	< 1
25	F	62	IRL	Posterior	P	27	M	ADC	Well differentiated ADC	wt	wt	wt	< 1

*SRL* superior right lobe, *ML* medial lobe *IRL* inferior right lobe, *SLL* superior left lobe, *ILL* inferior left lobe, *P* peripheral, *M* medial, *NS* non-solid, *M* mixed, *wt* wild type

**Table 2:** Exhaustive radio-morphologic and molecular data on the evaluated NS nodules approached through FNA bioptic procedures

ID#	Gender	Age at diagnosis (yrs)	Lobe	Segment	Site	Diameter (mm)	Density M/NS	Cytology	Histology	Architectural pattern	EGFR	ALK/EML4 ROS	PDL-1 (%)
1	M	80	ILL	Apical	P	15	NS	ADC			wt	wt	< 1
2	M	72	SLL	Apical	P	35	M	Well differentiated ADC	Well differentiated ADC	Lepidic (90%) acinar (10%)	wt	wt	< 1
3	F	70	SLL	Apical	P	9	NS	ADC			wt	wt	< 1
4	M	72	SRL	Posterior	P	23	M	ADC	Partially differentiated ADC	Lepidic (50%) paillary (30%) acinar (20%)	wt	wt	< 1
5	M	71	ILL	Apico-posterior	P	40	M	ADC	Partially differentiated ADC	Acinar (40%) papillary (40%) lepidic (20%)	wt	wt	< 1
6	M	68	IRL	Posterior	P	30	M	ADC			wt	wt	< 1
7	M	65	IRL	Apical	P	16	NS	ADC			wt	wt	< 1
8	M	70	SLL	Lingular	P	13	M	ADC	Mucinous ADC, partially differentiated	Acinar (75%) lepidic (20%) micropapillary (5%)	wt	wt	< 1
9	M	76	SLL	Apical	P	30	M	ADC			wt	wt	< 1
10	F	63	ILL	Apical	P	20	M	Atypia /ADC	Well differentiated ADC	Acinar (100%)	wt	wt	< 1
11	F	70	SRL	Posterior	P	15	M	ADC	Well differentiated ADC	Acinar (40%) papillary (40%) lepidic (20%)	wt	wt	< 1
12	M	67	SRL	Posterior	P	24	M	ADC	ADC, partially differentiated (G2)	Lepidic (35%) papillary (35%) micropapillary (30%)	wt	wt	< 1
13	F	65	SRL	Posterior	P	12	M	ADC	ADC, partially differentiated (G2)	Acinar	wt	wt	< 1
14	F	71	IRL	Apical	P	10	M	ADC	ADC, partially differentiated (G2)	Acinar, lepidic	del ex 19	wt	< 1
15	M	67	SRL	Posterior	P	10	M	ADC	Well differentiated ADC	Acinar, lepidic	wt	wt	< 1
16	F	81	IRL	Apical	P	32	NS	ADC	ADC	acinar, lepidic	wt	wt	< 1
17	M	78	SLL	Apical	P	28	NS	ADC	ADC, partially differentiated (G2)	Lepidic, micropapillary	wt	wt	30
18	F	69	SRL	Posterior	P	17	M	ADC TTF1+	Minimally invasive, partially differentia	Ltepdi dAiDc,Cfocus invasive acinar (5 mm)	wt	wt	< 1
19	M	72	IRL	Postero-basal	P	30	M	Well differentiated ADC	ADC, partially differentiated (G2)	Papillary	wt	wt	< 1
20	F	71	ML	Basal	P	43	M	ADC	Well differentiated ADC	Lepidic, papillary	wt	wt	< 1
21	M	67	SRL	Apical	P	30	M	ADC TTF1+			wt	wt	2
22	M	75	IRL	Apical	P	13	M	ADC			wt	wt	5
23	F	75	IRL	Latero-basal	P	15	M	ADC			wt	wt	< 1
24	M	75	SLL	Apico-posterior	P	15	M	ADC	ADC, partially differentiated (G2)	Lepidic, acinar	L858R	wt	< 1
26	M	68	SLL	Superiore	P	12	NS	ADC			wt	wt	< 1
27	F	70	SRL	Dorsal	P	14	NS	ADC			wt	wt	< 1
28	M	71	IRL	Apical	P	15	NS	ADC			wt	wt	< 1
29	M	71	SRL	Medial-basal	P	13	NS	ADC	ADC, partially differentiated (G2)	Acinar, intense inflammatory infiltrate	wt	wt	< 1
31	M	78	ML	Medial	PM	25	NS	ADC			wt	wt	< 1
32	M	62	SLL	Medial	P	18	NS	ADC	Poorly differentiated ADC (G3)	Solid, micropapillary, linfoinvasive	wt	wt	< 1
33	M	74	SRL	Medial	P	22	NS	ADC	Partially differentiated ADC	Acinar, micropapillary	wt	wt	10
34	M	75	SRL	Superior	P	34	NS	ADC			wt	wt	< 1
35	F	76	SRL	Apico-posterior	P	36	NS	Mucinous ADC			wt	wt	< 1
36	F	65	IRL	Apical	P	19	NS	ADC			wt	wt	< 1
37	M	73	SRL	Apico-posterior	P	27	NS	ADC	ADC, partially differentiated (G2)	Lepidic (40%), papillary (30%) micropapillary (30%)	wt	wt	< 1

*SRL* superior right lobe, *ML* medial lobe *IRL* inferior right lobe, *SLL* superior left lobe, *ILL* inferior left lobe, *P* peripheral, *M* medial, *NS* non-solid, *M* mixed, *wt* wild type

### CT-guided biopsy procedure

CT-guided biopsies were performed using different multidetector scanners. The biopsies were performed both with conventional and fluoroscopic approach, depending on the nodule characteristics and location. During a fluoroscopic biopsy the operator stayed close to the patients inside the gantry room; the table could be moved using a joystick and the image can be created in real time using a pedal. Images can be obtained with a frame rate up to 3 frames/s. Generally, only in the first step of the procedure a real fluoroscopic vision was needed in order to study the movement of the nodule, the ribs and the diaphragm (for basal nodules); in the latter stages only one-shot images were needed to check the position of the needle compared to the nodule. The gantry could be tilted up to + or − 22° to avoid ribs or vessels. Conventional CT approach is characterized by short spiral acquisition to check the position of the needle, performed without the presence of the operator inside the gantry room. Spiral acquisition allowed progressive adjustments of the needle position, performed in-bore, between different spiral acquisitions.

Dose-length product of a biopsy procedure was generally around 100 mGy*cm (DLP) ranging from 50 to 500 mGy*cm on the patients. The dose to the operators varied on the same range and never exceeded the regulatory limits for eye-lens, hands and body even for operators that performed up to 200 procedure a year (generally no less than 4 procedure a week). Among the personal protective equipment for fluoroscopic procedure, there were lead apron, glasses and gloves.

CT-guided biopsies were performed both as fine-needle aspiration biopsy (FNAB) and core biopsy (CB). FNAB was performed with chiba needles (HS Diva, HS, Italy) ranging from 24G to 20G (several needles had a 24G tip and 22G body in order to be less invasive on the pleura but at the same time offering a wider diameter to avoid clogs). The length of the needle could be 10 or 15 cm. The aspiration and the to-and-fro movement ensured the capillary draining of the material inside a small syringe connected to the needle. No automatic aspiration pistols were used since the space inside the gantry is limited especially on the lateral sides of the patient; we avoided automatic aspiration especially for non-solid nodules since a close control of the aspiration strength avoid exceeding in aspiration which results in an alveolar haemorrhage. When the tip of the needle was inside the lesion the aspiration time could be modulated by the operator on the basis of the quantity and quality (perceived by the operator) of the material aspirated. The tip of the needle was small enough to be directed and inserted on the solid portion of mixed nodules in order to sample the locations more likely to release good quality material with a higher percentage of neoplastic cells. Since in non-solid nodules only a small amount of cells plastered the alveoli surface one of the advantages of the FNAB was to reach different portion of the nodules in order to “enrich” the material by sampling different areas of the nodule. Generally, we tried at least 3 different location; but it is hypothetically possible to reach every portion of the nodule with the same needle insertion. Core-biopsy needle were semi-automatic models ranging from 20G to 16G (BiopsyBell, BPB Medical, Italy and Precisa, HS, Italy), which can retrieve samples of 10 or 20 mm in length. The approach was similar to that of FNA biopsy, but the retrieval of the micro-histologic sample is performed with a two-step procedure: advancement of the needle inside the lesion and firing of the mechanism.

Complication rates were extremely low (around 25% of any kind and grade of complication; less than 7% of clinically relevant complications and 3% of complications requiring hospitalization). Material obtained from FNA was fixed in formalin in order to obtain a cell/cyto-block for the pathologist; material obtained from CBN was also fixed in formalin after minimal manipulation to remove it from the needle cradle.

All patients who were routinely assuming warfarin or platelet antiaggregants were asked to stop their therapies and to shift to low molecular weight heparin that was then suppressed the day before the procedure. Platelets counts at baseline were within normal range for all patients.

### Cytological, histological and molecular techniques

A rapid on-site evaluation (ROSE) cytopathology service was not available. In case of FNA, no aspirative smear was assessed, and all the obtained material was immediately processed with 10% formalin fixation and paraffin embedding to obtain a cell block. It is widely accepted that cell block technique increases diagnostic accuracy with efficient results [17–19]. For both cell block and histologic specimens, haematoxylin and eosin (H&E)-stained sections were evaluated by a pulmonary cytopathologist; immunohistochemical stains were performed whenever required for the precise characterization of neoplastic cells [20, 21]. According to recent guidelines [22], non-small cell lung cancer (NSCLC) cytological samples were submitted to immunohistochemical assay of PD-L1 expression levels using a laboratory-developed test designed to optimize the use of anti-PD-L1 22C3 antibody (Dako) on the Omnis autostainer (Dako) with Envision FLEX (Dako) revelation system. Tumor proportion score (TPS) was evaluated according to published guidelines [23]. Lung resection specimens were fixed in formalin and embedded in paraffin for routine histopathological evaluation; histopathological patterns of ADC were assed and quantified with 5% increments according to most recent guidelines (archives 2011). In cases diagnosed as primary lung adenocarcinomas (ADC) mutational analysis of the coding sequence of *EGFR*-TK domain and evaluation of *ALK/EML4* and *ROS1* translocations were performed as already published [24–26] on both cytologic and surgical specimens when required by the oncologist.

### Statistical analysis

Descriptive statistics has been obtained for all variables assessed in the study population. Groups have been compared by means of parametric or nonparametric tests for quantitative variables and Pearson’s Chi-square for categorical variables.

## Results

### Clinical and radiological features of NSNs

We identified 62 patients who were diagnosed at our Institutions with lung malignant NSNs. Of them, 26 were females (41.9%) and 36 (58.1%) males; the mean age at diagnosis was 69, 35 years. Thirty-four patients were never smokers, while 14 were past smokers and only 4 was a current smoker. Smoking history was unknown in 9 cases. Twenty-five (40.3%) out of the 62 evaluated patients referred a previous diagnosis of cancer and were in remission at time of enrolment; one patient was assuming immunosuppressive drugs due to previous heart transplantation. Forty out of the 62 lesions were in the right lung, 30 in the upper lobe, 8 in the lower and 2 in the middle one, respectively. Seventeen were in the left lung, 10 in the upper lobe, 6 in the lower and 1 in the lingula, respectively. Overall, in 26 cases consisted of basal parenchymal lesion lesions, more difficult to be approached. Based on nodule localization, the procedural approach was posterior or anterior. Most lesions occurred peripherally within the lung parenchyma (48 patients, 77.4%); while 13 cases featured peri-mediastinal nodules. Twenty-nine (46.77%) of the analysed nodules displayed a contact with the pleural layer. Of them, 4 nodules grew in contact with the fissure, in 16 a small peduncle was detected whereas the remaining 9 featured a wide interface with the visceral pleura. The vast majority of nodules displayed a round/ovalar shape with irregular margins. The aerated lung run of the needle was between 1 mm and 60 mm respectively, the minimal distance from middle-large veins and arteries (segmental, lobar and main pulmonary) was of 3 mm. In 37 (59.6%) cases, nodules had mixed or semi-solid appearance, the remaining were pure non-solid. All but seven lesions were small, displaying a diameter ≤ 3 cm; in only two case the diameter was of 5 cm and 6 cm, respectively.

Thirty-seven out of all the patients underwent transthoracic FNA TC-guided biopsy (needle: 24 gauge) leading to a diagnosis of NSCLC (ADC) in 31; in six cases, the diagnosis was inconclusive (suspicious for neoplastic cells); in 3 cases out of the latter a second FNA was performed, leading to a conclusive ADC diagnosis. In 25 patients transthoracic TC-guided CNB (needle: 18 gauge) was performed and allowed the diagnosis of NSCLC (ADC) in all cases.

The diagnosis of lung ADC was reached in all cases on the sole basis of cell morphology and architecture on H&E stained slides derived from both FNA and CNB tissue samples; immunohistochemical staining was required in 3 cases to exclude metastasis form a previous extrapulmonary ADC.

Overall fourteen out of the 62 cases early minor complications (22.5%). In detail, among the 37 nodules biopsied through FNA, minor complications aroused in 6 cases (9.6%), one patient presented mild haemoptysis rapidly solved after tranexamic acid infusion; whereas in the remaining five a small pneumothorax could be demonstrated after the procedure. In the cohort of the 25 nodules reached through by CNB, 8 (12.8%) cases presented early small pneumothorax demonstrated after the procedure. Major complications regarded the occurrence of complete pneumothorax which was managed by chest tube insertion and drainage in one case and in two case in the cohort of FNA and CNB, respectively. No late complications or pleural or bleeding/haemothorax occurred in the cohort of FNA procedures whereas in two cases who underwent CNB a minimum haemothorax occurred, without any further therapeutic management required. No late complications occurred. All the 62 patients did not refer significant pain during and after procedure (Numerical Rating Scale NRS ≤ 3).

### Morphologic and molecular profiling of NSNs

Based on early disease stage, all the enrolled patients were addressed to surgical resection of lung cancer nodules to exclusive radiotherapy or to exclusive radiotherapy.

In 20 out of the 37 cases diagnosed through FNA, surgical resection was conducted at our Institution. The latter allowed the unique opportunity to perform a complete morphological and molecular match between the FNA-derived samples and their corresponding removed tissue. Surgical resection confirmed the cytological diagnosis of invasive or minimally invasive ADC in all the 20 cases. One case was in situ, 4 were minimally invasive and 15 invasive. The predominant histopathological pattern was lepidic in 7 (35%) cases, papillary in 6 (30%), acinar in 3 (15%), and solid in 1 (5%); 3 cases (15%) had an equivalent proportion of acinar and papillary, acinar and lepidic, and papillary and lepidic components. Seventeen cases showed the coexistence of more patterns: 4 acinar and lepidic, 1 each lepidic and papillary, papillary and acinar, solid and micropapillary, 3 lepidic acinar and papillary, 2 acinar papillary and micropapillary, two lepidic papillary and micropapillary, 2 lepidic, acinar, papillary and micropapillary. Three cases showed a homogeneous pattern, 1 acinar, 1 papillary and 1 lepidic. Overall, a lepidic component was present in 13 cases, acinar in 14, papillary in 12, micropapillary in 8, and solid in 1. No specific pattern could be recognized in FNA cytological samples but unequivocal papillae in the case with exclusive papillary architecture. The occurrence of unsatisfactory FNA samples was not associated with any specific histological pattern. No acinar components in non-solid nodules as acinar components could account for solid portion of the mixed subsolid-nodules.

PD-L1 immunostains could be evaluated in 30 cytological samples, while the remaining 7 did not reach the cellularity threshold for evaluation. TPS was < 1% in 26 cases,  > 1%/< 50% in 3, and > 50% in 1. All surgical samples showed TPS < 1%. Of the 20 cases that could be evaluated on both cytologic and surgical samples, 18 were concordantly TPS 0, and 2 showed TPS > 1% < 50 on the biopsy samples.

*EGFR* mutations were identified in 2 of the 28 cases (7%) with adequate material (one exon 19 deletion and L858R); no case showed *ALK/EML4* or *ROS1* translocations.

Eight of the 25 cases diagnosed through CNB were patients that after the biopsy decided to continue the diagnostic-therapeutic path in other structures. In eleven of the 25 cases, surgical resection was performed. Surgical resection confirmed the cytological diagnosis of invasive or minimally invasive ADC in all 11 cases. The predominant histopathological pattern was acinar in 8 cases, papillary in 2 and acinar in 1.

PD-L1 expression could be evaluated in 22 samples, while the remaining three samples did not reach the cellularity threshold for evaluation. TPS was < 1% in 15 cases,  > 1%/< 5% in 4 cases, > 5%/< 50% in 2 cases, > 50% in 1 case.

*EGFR* mutations were identified in 4 of the 25 cases (16%) with adequate material (one exon 19 deletion, L858R, L861Q, G719A); no case showed *ALK/EML4* or *ROS1* translocations.

Overall, being the population evaluated in the study homogenous, the Chi squared analysis (with degree of statistical freedom k = 1) of two distribution regarding the *EGFR* mutational profiling, allows us to reject the null hypothesis that the two groups FNA biopsy and CNB biopsy) are the same, whereas regarding the PD-L1 expression the two frequencies are coherent with the expected one. In other words, by comparing the two bioptic techniques, FNA and cell block cytology result in an *EGFR* mutational frequency lower than the expected one. In conclusion, cell-block cytology appears to be adequate for IHC stain but not in case of DNA direct sequencing.

## Discussion

Despite relevant progresses in personalized approach to lung cancer, many aspects remain to be clarified. One of the most important issue is that lung cancers have different pathologic features. In this perspective non-solid malignant lung nodules define a still obscure neoplastic entity. Although many reports are already available regarding diagnostic approach to NSNs, fewer data exist in terms of their molecular and pathological characterization. We retrospectively reviewed a relevant cohort of NSN that were first confirmed as malignant on CT-guided FNA and were subsequently resected. Importantly, we further analysed a corresponding cohort of NSNs which were approached by core biopsy. This gave us the unique opportunity to perform exhaustive pathological and molecular profiling of tumor material on both cyto- and histological specimens focusing on two complementary goals: (i) the characterization of the histopathological and molecular profiles of non-solid neoplastic growth; (ii) the validation of a panel of molecular markers on cytological samples as consistent (predictive) to those of the whole lesion.

The findings of our study indicate that CT-guided FNA is a safe, feasible and accurate procedure as CB for the diagnosis of NSNs and mixed neoplastic nodules. In 83% of cases, a conclusive diagnosis was reached on the first sampling, while the remaining were classified as suspicious, which in 2 cases lead to surgical resection with intraoperative confirmation of neoplastic nature, and in 3 to a second CT-FNA with conclusive diagnosis. As expected, and coherently with the most published data, all malignant NSNs where diagnosed as ADC. By comparing data obtained on the two cohorts, several aspects deserve to be underlined. The first is that the development of the cell block method, a complementary approach to conventional FNA cytology, is responsible for the 100% accuracy of tumor histotype definition on FNA cytology. The possibility to submit cell block sections to IHC staining also allows a conclusive differential diagnosis in patients with a previous history of extrapulmonary ADC. However, the most relevant advantage associated with cell block is the possibility of using these samples for molecular subtyping and definition of immunotherapy response predictive markers. Reflex PD-L1 ICH stain is recommended, considering that 4 of the 7 (19% of the total) FNA samples with insufficient cellularity were from cell block recuts obtained after molecular analysis.

As far as histomorphology is concerned, most cases showed mixed patterns as generally reported; acinar, lepidic and papillary architectures were almost equally common in different combinations, but lepidic pattern were more frequently predominant. No specific architectural pattern could be recognised on FNA. PD-L1 immunostains documented the predominance of low/negative TPS, with high concordance between FNA and CB and corresponding surgical samples. High TPS was observed in only 1 FNA sample and 1 CB sample, both not submitted to surgery. Low PD-L1 expression rates have previously been documented in in situ and minimally invasive ADC (0%), and in ADC with lepidic (0%) and acinar (36%) components, while papillary ADC scores are intermediate [27–29]. Similarly, studies aimed at correlating PD-L1 expression with CT scan features documented that PD-L1 was negatively associated with the presence of ground glass opacities surrounding the tumor nodule [30, 31]. No explanations have been provided so far for this phenomenon; it can be hypothesized that lung ADC with NSN pattern and predominant in situ (i.e. lepidic) components represent the first steps in tumor progression, which have not yet triggered adaptive immune-resistance through T cell activation and ifn-gamma secretion, and/or have not accumulated a significant rate of mutations and neoantigen production, or that they belong to the infiltrated-excluded category of tumors [32].The negative prediction of response to immunomodulating therapy underlines the importance of rapid surgical treatment of these lesions.

The vast majority of patients did not refer a smoking history. Despite that, a very low rate of actionable mutations was identified in our cohort: no alterations were detected in *ALK* and *ROS1* genes, *EGFR* mutational frequency was lower than expected in this population (7% vs ≈ 15% of the expected one) in the FNA cohort, whether by sequencing CBN-derived samples the mutational frequency is coherent to the expected (16%). While the first result is consistent with literature data reporting a low rate of ALK rearrangement in papillary or lepidic predominant, in situ and minimally invasive ADC [33, 34], most published studies obtained in Asian populations agree on the high prevalence of *EGFR* mutations in these ADC subgroups [32, 33], and more generally in nodules with ground-glass CT features [35]. Within the limit of the cohorts analysed, these data suggest different tracks leading to the development of solid and non-solid tumors.

## Conclusions

To the best of our knowledge, this is the first report which aims at comparing–in non solid adenocarcinomas—two CT-guided diagnostic approaches. Interestingly, they result comparable regarding ICH staining, as demonstrated by PD-L1 expression analysis that is coherently low in matched cytologic, histologic and surgical samples. Notably, in case of DNA direct sequencing, FNA cell-block specimens seem to be not fully performing mainly due to the low DNA mass. It is conceivable that DNA extracted from cell blocks should be more efficiently processed through next generation sequencing in high-throughput laboratories, but more data are required to validate this suggestion. Overall, at least in case of NSNs, the diagnostic approach should be decided mainly by taking under consideration the further process of the obtained sample.

## Data Availability

The datasets used and/or analysed during the current study are available from the corresponding author on reasonable request.

## Abbreviations

ADC: Adenocarcinoma
FNA: Fine needle aspiration
CNB: Core needle biopsy
CT: Computed tomography
NSN: Non solid nodule
DLP: Dose-length product
PD-L1: programmed death ligand 1
TK: Tyorisine kinase
TPS: Tumor proportion score
